# Adoption of Tobacco 21: A Cross-Case Analysis of Ten US States

**DOI:** 10.3390/ijerph18116096

**Published:** 2021-06-05

**Authors:** Shawna V. Hudson, Marin Kurti, Jenna Howard, Bianca Sanabria, Kevin R. J. Schroth, Mary Hrywna, Cristine D. Delnevo

**Affiliations:** 1 Department of Family Medicine and Community Health, Rutgers Robert Wood Johnson Medical School, Rutgers, The State University of New Jersey, New Brunswick, NJ 08901, USA; howardje@rwjms.rutgers.edu (J.H.); bianca.sanabria@rutgers.edu (B.S.); 2 Rutgers Cancer Institute of New Jersey, New Brunswick, NJ 08901, USA; delnevo@rutgers.edu; 3 Department of Sociology, Anthropology, Criminology and Social Work, Eastern Connecticut State University, 83 Windham Street Willmantic, CT 06226, USA; kurtim@easternct.edu; 4 Rutgers Center for Tobacco Studies, Rutgers University, New Brunswick, NJ 08901, USA; schrothk@rutgers.edu (K.R.J.S.); mary.hrywna@rutgers.edu (M.H.); 5 Department of Health Behavior, Society & Policy, Rutgers School of Public Health, Piscataway, NJ 08854, USA

**Keywords:** tobacco, policy, e-cigarettes, health behavior

## Abstract

Despite the recent push for Tobacco 21 legislation in the US and the national adoption of Tobacco 21, there is a paucity of data on the process of policy adoption. To explore the key factors that served as facilitators or challenges to the passage of state T21 laws that apply to the sale of all tobacco products to anyone under 21 years of age, we conducted a comparative, cross-case study in ten states that adopted Tobacco 21 between 2016 and 2019. Stakeholders from selected states were identified via snowball sampling, and interviews were conducted from November 2018 to March 2020. Three primary factors emerged as facilitators to the passage of state T21 laws: (1) increased attention on e-cigarettes as the product driving an overall increase in youth tobacco use and depiction of an “e-cigarette epidemic”, (2) having at least one influential policy entrepreneur or champion, and (3) traction from other states or local municipalities passing T21 legislation. Challenges to T21′s success included (1) influence of the tobacco industry, (2) the bill’s low ranking among legislative priorities, and (3) controversy among advocates and policymakers over bill language. As e-cigarette rates spiked, T21 bills became legislative priorities, traction from other successful efforts mounted, and ultimately, the tobacco industry flipped from opposing to supporting T21 laws. Despite these favorable headwinds, advocates struggled increasingly to pass bills with ideal policy language.

## 1. Introduction

Nearly 90% of tobacco users start before age 18, and nearly 99% start before 26 [1]. Tobacco 21 (T21), a national campaign aimed at raising the minimum legal sale age (MLSA) for all tobacco products in the United States to 21 was designed to decrease access at retail and limit the social channels through which youth get tobacco products, with the goal of preventing or delaying tobacco initiation [2,3,4]. In March 2015, a seminal Institute of Medicine (IOM) report described the public health implications of raising the MLSA for tobacco products and concluded that a national T21 law would result in an additional 12% decrease in smoking prevalence and a 10% decrease in smoke-related deaths [5]. While a fairly recent tobacco control initiative, momentum around raising the MLSA has grown over the past decade. T21 laws were first passed primarily in localities, then the state level, up to 19 states and DC before finally culminating in Congress raising the MLSA to 21 in December 2019.

The few case studies examining local T21 adoption [6,7] point to varied opposition from diverse stakeholders. In Needham, Massachusetts, the first town in the US to raise its MLSA to 21 in 2003, opposition came from local and national stakeholders (e.g., local policymakers and merchants, national retail chains) with little tobacco industry influence [7]. That changed in 2013 when New York City passed the nation’s second Tobacco 21 law [7,8], with considerable pushback from the tobacco industry [7]. These local case studies also point to factors that facilitate T21 adoption. In Missouri, the simplicity of T21 contributed to its ease of adoption at a local level [6], whereas smoke-free air laws, which are not aimed exclusively at youth, were perceived as more complex and controversial [6,7,8,9].

Given the relatively recent push for T21 legislation, only two studies have examined the passage of state-level T21 laws. An analysis of state T21 laws passed by July 2019 described laws that varied widely across several recommended components [10]. A more recent analysis of all state- and territorial-level jurisdictions with T21 laws enacted by 20 December 2019 confirms wide variation in policy language [11]. Moreover, compared to laws passed earlier, T21 laws enacted in 2019 were more likely to contain negative policy language or clauses favored by the tobacco industry, including youth tobacco purchase, use, or possession (PUP) penalties, military exemptions, phase-in periods of one year or more, and preemption clauses [11]. There is consensus among tobacco control advocates that PUP laws, which penalize youth for purchasing, using, and possessing tobacco products, are ineffective at reducing youth tobacco use and inequitably enforced [12,13]. Two other popular provisions to T21 laws included allowances for members of the military and those who could purchase tobacco under the previous law (e.g., grandfathering or phase-in periods), which undermine the public health impact of the law by excluding specific groups at risk for tobacco initiation and progression to regular smoking [14,15,16]. The tobacco industry has historically pursued statewide preemption—when a state restricts or eliminates the authority of local governments to pass laws that differ from state law—as a tool to block, weaken, and delay progressive tobacco control policies, youth access laws among them [17].

No studies have examined the dynamics of T21 policy adoption across more than one state or locality. While the new federal law sets a uniform MLSA, it relies on states to implement the methods necessary to enforce the law. Since the passage of the federal law, 14 more states passed T21 laws in 2020. A better understanding of the policy process across several states may be valuable in informing state and local authorities that will now need the tools to effectively implement a T21 law to reduce youth access to tobacco. Thus, we conducted a cross-case analysis of 10 states that successfully passed T21 legislation between 2015 and 2019 and sought to identify the main factors that helped bills to gain momentum as well as the most salient obstacles to bill passage.

## 2. Materials and Methods

**Figure 1:**
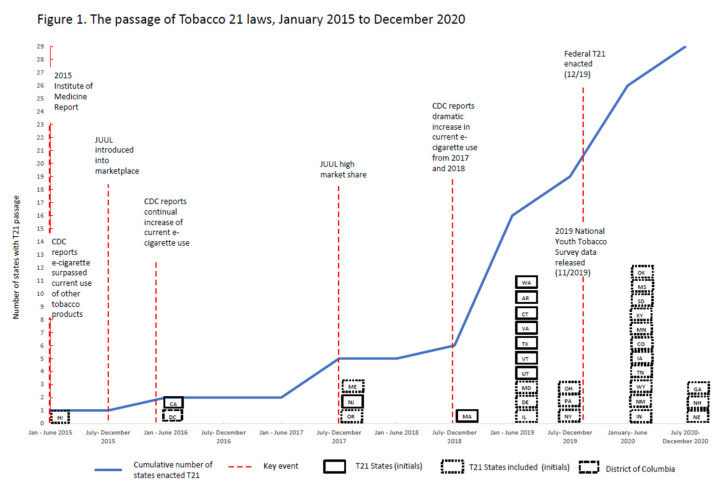
The passage of Tobacco 21 laws, January 2015 to December 2020.

We conducted purposive sampling to construct case studies of 10 states. States which had passed T21 legislation were selected based on recommendations from an external advisory board that advocated for a maximum variation sampling approach to facilitate exploration of the relevance of potential variations in policy context, geography, and timing of the bill’s passage (e.g., early adopter vs. early majority) (see Figure 1).

Stakeholders from selected states were identified via snowball sampling, beginning with recommendations from our advisory board. Interviews were conducted from November 2018 to March 2020 with policymakers, health department workers, and advocates from national and regional health organizations (see Table 1). All stakeholders were interviewed about a particular state; this was also the case for advocates who worked on campaigns in multiple states. Potential participants were invited via email and/or telephone. Of the 92 individuals responding to our invitation, 78 participated, 5 declined, and 9 were unresponsive after initially expressing interest (85% participation rate). This is consistent with the Standards for Reporting Qualitative Research (SRQR) [18].

Using the multiple streams framework (MSF) [19,20] as the conceptual model, we focused on understanding how T21 became relevant for legislative agendas when three independent streams—the problem, the policy, and the politics stream—converge to open up a policy window of opportunity. This convergence is influenced by policy entrepreneurs (i.e., special interest groups) who advocate and actively participate in defining the problem and presenting the “solution” (i.e., policy) to policymakers (politics) [19,20,21,22]. Major topics covered in the interviews included the following: (1) the chronology of the state’s T21 campaign and legislative process (problem), (2) the interviewee’s role and the roles of other individuals and organizations that were involved (politics), (3) factors that helped T21 to gain traction in their state (politics), (4) factors that slowed or impeded Tobacco 21′s success (politics), and (5) the final legislation and lessons learned through the process (policy and politics). Additional prompts sought stakeholders’ perspectives on the role that the news media played in the campaigns, education and enforcement efforts in the implementation of the laws, and speculations of the next chapter in the T21 story. Two interviewers (JH and MK) conducted digitally recorded, semi-structured telephone interviews that ranged in length from 30 to 90 min. All interviews were transcribed verbatim. Participants were offered a USD 20 gift card as an incentive.

Data analyses followed a collaborative, interpretive process of “immersion/crystallization” [23,24]. Using the same inductive reasoning that characterizes grounded theory [25,26], immersion/crystallization comprises iterative cycles of reading and reflecting on the data until themes begin to emerge. This process was conducted on a rolling basis and occurred simultaneously with data collection. The team met weekly to identify emerging patterns and determine the point of saturation (the point at which interviews no longer yield new information) for each state case [27]. Themes that emerged within and across cases informed codebook development that 3 coders (MK, BS, JH) utilized in ATLAS.ti version 8 (Scientific Software Development GmbH, Berlin, Germany). Initially, the coders coded 3 interviews together to calibrate code definitions resolving disputes by consensus, then 2 coders (BS and MK) coded the remaining data, with weekly meetings with the team to check for consistency. At the conclusion of data collection, the analysis team engaged in another round of immersion/crystallization with the coded data, resulting in a synthesis of facilitators and challenges most salient in each state.

## 3. Results

### 3.1. Facilitators

Stakeholders described three primary factors that served as T21 facilitators: (1) policy diffusion from other states and/or local municipalities passing T21, (2) having a strong champion or policy entrepreneur, and (3) the rise of the e-cigarette epidemic (Table 2).

#### 3.1.1. Policy Diffusion

In some states, local diffusion predated the introduction of the state bill (i.e., vertical diffusion). In Massachusetts, more than 230 localities, including Boston, passed T21 laws before the state, demonstrating the popularity of the issue to state legislators. In Utah, stakeholders described the local activity as a catalyst prompting the state to consider T21 for statewide uniformity. Stakeholders in New Jersey noted that the passage of a local ordinance in the capital was influential: “I think probably a big turning point for us was when Trenton passed their ordinance. I think we started to get a little bit more attention to this and that’s when it really started moving...” (NJ 01, National advocacy). Horizontal policy diffusion also fueled momentum in other locations. A stakeholder in Connecticut commented, “What’s happening around the country and then more towns and cities taking action, I think, helped.” (CT 01, National advocacy) Some indicated this trend motivated them to pursue the cause so as not to be left behind.

#### 3.1.2. Policy Entrepreneurs

All stakeholders described policy entrepreneurs as crucial for passing T21. While in all states, legislators served as the primary policy entrepreneurs, in some states, they were joined by physicians to facilitate the movement of policy proposals. In Arkansas, the primary policy entrepreneur was a legislator who was also a physician. While most policy entrepreneurs were described in terms of passion, willingness to work hard, and persistence in the face of opposition, others were recognized for strategic vision. In Texas, the bill author garnered bipartisan support, recognizing that the governor and lieutenant governor might not approve the bill if it were perceived as a Democratic effort. He worked diligently to recruit “several prominent Republican senators as sponsors of the bill, so it had a very well-represented, almost evenly split between Democrats and Republicans in terms of sponsors for the bill and the ultimate vote….” (TX 01, National advocacy), demonstrating that policy entrepreneurs played a critical role in building support for state-level T21 laws from other decision makers and influenced the convergence of the problem, policy, and political streams.

#### 3.1.3. The Rise of the E-Cigarette/Vaping Epidemic

As described in MSF, public policies occur when political entities want solutions to issues that they perceive as a problem [22]. Stakeholders described the recognition of an “e-cigarette/vaping epidemic” as a focusing event in gaining legislative and advocacy support for T21 as a solution to this problem. Nevertheless, the increase in youth e-cigarette use was not consistent over time; it was punctuated by surges, flurries of media coverage, and other events. States such as California and New Jersey passed T21 relatively early (Figure 1), while a number of other states passed their laws *after* 2018 data revealed a dramatic surge in youth e-cigarette use. California was the second state, after Hawaii, to pass T21 in May 2016. One advocate credited increasing e-cigarette use, which went up in 2014 and 2015 nationwide, as indicators of a potential problem, creating “a moment” of political opportunity for Tobacco 21 in California (CA 01, Regional advocacy).

However, the release of data from the 2018 National Youth Tobacco Survey (NYTS) in November 2018 reflecting a 75% increase in current e-cigarette use among youth was a powerful impetus for action and recognizably preceded policy change in several states. Virginia and Arkansas introduced bills in early 2019 and passed them in the fall. A regional health advocate from Arkansas suggested that this was “...probably the main catalyst. I think that certainly legislators there recognized this problem, heard from their constituents…and felt like the T21 policy might be a good solution to try to tackle that.” (AR 01, National advocacy) Stakeholders in other states also described this as particularly salient. Some told stories about how vaping “definitely helped our legislators understand the urgency of addressing the problem.” (WA 01, National advocacy) Moreover, participants described the persuasive power of youth who had been trained by advocacy organizations to testify before the legislatures. Rallies were organized as a forum for youth to publicly advocate for T21. Youth advocacy was described by some stakeholders as pivotal in adding to legislators’ knowledge about the severity and implications of rampant e-cigarette/vaping use.

### 3.2. Challenges

While the states highlighted in this study passed T21 legislation, there were significant challenges that impacted the strength of the laws. The most pervasive challenges noted can be categorized into three areas: (1) the influence of the tobacco industry, (2) perceptions of the bill’s low rank among legislative priorities, and (3) controversy among advocates and policymakers over the bill language (see Table 2 and Table 3).

#### 3.2.1. Industry Influence

In early adoption states, participants described the tobacco industry as a major opponent to T21. In some cases, the tobacco industry (e.g., New Jersey, Washington, Massachusetts, and Connecticut) actively opposed the bill and was overt in their effort to lobby legislators and energize retailers to voice concern over revenue loss from potentially reducing tobacco sales. In other states—Utah, Virginia, Arkansas, and Texas—industry influence was described as behind-the-scenes, which made the degree of their obstruction more difficult to gauge.

Tobacco industry influence shifted as T21 gained momentum. In Texas, an advocate noted, “…Altria made a decision in 2018 at the national level not to oppose T21, so their lobbyist here in 2019 had begrudgingly adopted that and dropped their opposition.” (TX 02, National advocacy). Industry support ranged from writing letters of support for increasing the MLSA to purportedly proposing and/or drafting the legislation. The bills supported by the industry were described by advocates as weaker and often included preemption clauses. In Virginia, tobacco control advocates described being “blindsided” by the tobacco industry urging legislators to propose T21. According to one participant, “This was not on anybody’s radar. It really came out of the blue...It was not anything that any of the tobacco control people brought forth.” (VA 01, Health departments).

Some advocates speculated that the change in industry stance was related to managing their public image and using it as a strategy to insert other laws that would limit future tobacco control policies. Indeed, both Virginia’s and Utah’s laws penalize purchase, use, and possession by the underage consumer and exempt military personnel [29,30]. Advocates also pointed to the industry strategically promoting the introduction of bills at the end of a legislative session (e.g., Virginia), a strategy often calculated to minimize opportunities for opponents to influence a bill’s text. In this way, the tobacco industry also played a role as policy entrepreneur, although their investment in shaping and supporting T21 legislation came late in the process.

#### 3.2.2. Low Ranking among Legislative Priorities

A difficult challenge for Massachusetts, Washington, Virginia, and Arkansas was that T21 was initially not seen as a priority. National advocacy organizations tended to be reluctant to engage on T21 without hard evidence to support the campaign. The release of the IOM report provided the evidence some stakeholders needed to get on board with T21. For example, one advocate described the IOM report as “the key that unlocked our [organization’s] enthusiastic support for T21.” (WA 01, National advocacy) In other cases, advocates resisted T21 out of concern that it might be a substitute, rather than a supplement, to a comprehensive approach to tobacco control. That is, some advocates feared that if T21 passed, the legislature would consider themselves “done” with tobacco control, making it impossible to pass other tobacco control priorities.

Even after T21 had become a priority for advocates and public support for T21 had been consistently high, it was often not a priority for lawmakers [31,32,33,34,35,36]. Stakeholders in several states described the difficulty of getting T21 on the radar of legislators. “I think sometimes, tobacco in particular, especially in the northeast where we’ve made a lot of progress, people felt like we had done enough, and did we really need to do this? So the sense of urgency at times…was a factor and a barrier for us.” (MA 01, National advocacy)

#### 3.2.3. Controversy over Bill Language

In states such as Washington, Virginia, Utah, and Arkansas, controversy over the language included in the bill was a major challenge. Advocates tended to argue against language that preempted local jurisdictions within the state from passing other types of tobacco control laws, PUP laws, and military exemptions. The degree to which advocates considered these provisions to be “deal breakers” changed over time. In Texas, for example, one organization of the tobacco control coalition withdrew support when preemption was added to the legislation. An advocate recounted, “[T]hey just could not support the bill as it passed. So they were out and they never came back on because that was the end.” (TX 03, Regional advocacy) Texas’ law also exempted members of the military [37]. Virginia’s law [29], which was supported by the tobacco industry before the advocates became involved, contains PUP provisions and a military exemption. One national advocate explained her organization’s opposition by highlighting that PUP provisions penalize youth instead of retailers and are ineffective at reducing youth consumption.

## 4. Discussion

This study examined how policy making dynamics interacted as 10 states pursued and passed T21. During the process, legislative champions were pitted against entrenched special interests, emerging scientific data and traction from successful jurisdictions elevated T21 above competing legislative priorities, and policymakers debated and refined their thinking on ideal policy solutions. Ultimately, the 10 states that we studied were among the 19 states and DC that passed T21 laws before T21 became US law. The 2015 IOM report enhanced the credibility and heightened the profile of the public health rationale supporting Tobacco 21 and momentum from local T21 laws. During this time, conventional policy-making patterns held true. Advocates educated lawmakers, and policy entrepreneurs made the case that T21 was an evidence-based policy that would prevent addiction and save lives.

Increases in youth e-cigarette use, and in particular increases associated with the rapid rise in sales of one e-cigarette brand, JUUL, track closely with the momentum for the T21 movement. The policy window for state T21 laws began to open when news reports in 2017 started documenting the rapid rise in JUUL [38]. While e-cigarettes were popular among youth prior to JUUL, it was primarily in the form of non-branded vapes or tank/mods [39]. Although JUUL was not the first major branded e-cigarette, it was without question the first major branded e-cigarette to strongly resonate with young people. Public attention regarding youth use grew, and the volume of news stories reporting on youth use more than quadrupled from 2017 to 2018 [38]. A focusing event happened in fall 2018 when CDC reported 2018 NYTS data that showed that youth e-cigarette use nearly doubled to 20.8% [40]. Despite strong public support for Tobacco 21, concerns over increased youth tobacco use reached unprecedented levels and catalyzed efforts to pass state T21 bills. This appears to be the window of opportunity—the critical point in time when the multiple streams of problem, policy, and politics converged to open the window for T21. In response to the alarming youth data, FDA Commissioner Scott Gottlieb instructed e-cigarette manufacturers to submit robust plans to address the widespread use of their products by minors or risk increased enforcement action [41]. Thus, the threat of FDA regulation provided powerful motivation to manufacturers to show they were serious about reducing youth access and altered the T21 policy landscape. Supporting T21 was one way to do so, and stakeholders reported how the 2018 data altered the industry’s positioning. By shifting to support T21 laws, the tobacco industry appeared to increase its influence, albeit in states that did not have longstanding tobacco control traditions, such as Virginia, Arkansas, and Texas. In these cases, lack of strong policy entrepreneurship in government or external advocacy organizations failed to counter the influence of the tobacco industry in shaping the problem and pushing for one policy solution over another. As a result, some more recent T21 laws contain clauses that the industry favored and tobacco control advocates consistently fought against, including preemption, militarily exemption, and PUP.

The themes identified in this case study are consistent with previous research on local, state, and national tobacco control policies that found that, among others, policy champions or change agents [7,42,43,44], engagement with youth advocates [7,45], supporting data, and other localities previously enacting the policy influenced adoption [42]. Past studies also suggest that the absence of supporting data may have hampered states which attempted to adopt T21 early [6,7], but in later years, the IOM report proved powerful enough to elevate the attention of T21 as a tool to reduce youth tobacco use.

We acknowledge several limitations to our study. While this study offers an analysis of the process to increase tobacco age of sale across multiple states, our study was limited to 10 states where T21 passed; thus, our data may only speak to the issues in states where T21 was ultimately successful. We did not include all states that adopted T21 or states where T21 failed. However, our study included cases where T21 was passed successfully after several failed attempts (e.g., New Jersey). In addition, our focus on state legislation or laws rather than federal or local legislation may have resulted in missing data on some important contextual factors. Although best attempts were made to identify the most relevant stakeholders to interview regarding T21, it is possible that some key informants were not available. For these reasons, generalizability may be limited. Furthermore, our study focus predates the federal law; 14 more states passed T21 after the federal law (See Figure 1).

## 5. Conclusions

Our case study of 10 states that passed statewide T21 laws demonstrated that while challenges and facilitators varied from state to state, there were common themes identified that impacted the adoption of T21 laws. The cultural and institutional context that fueled T21 legislative action including policy diffusion from other states and/or local municipalities passing T21, the work of strong policy entrepreneurs, and increased prevalence of e-cigarettes among youth. Countering the efforts to pass strong T21 laws were the influence of the tobacco industry, legislative capacity or will to deal with the problem, and controversy over policy language. Additionally, the factors identified in our study may continue to challenge and/or facilitate future efforts to increase tobacco age of sale or tighten existing laws. Even after nationwide support for T21, there may be barriers to adopting and implementing strong tobacco age of sale provisions at the state level, including industry efforts to weaken the effect of proposed legislation. As T21 legislation continues to expand to bring states in line with the federal law, this work can inform policy stakeholders who play a critical role in drafting new or strengthening existing state T21 laws to maximize public health. Ideally, new or updated state T21 laws will include strong policy language that can outline required protocols for compliance inspections, funding for enforcement, as well as monetary and licensing penalties for retailers [46,47]. State and local government involvement will be necessary to implement a T21 policy that will effectively reduce youth tobacco sales, and a model Tobacco 21 policy can minimize loopholes and ensure strong enforcement. Lastly, Tobacco 21 laws are the first step to limiting youth access to tobacco, but monitoring enforcement and compliance with T21 over time will also be important to evaluating and improving implementation.

## Figures and Tables

**Table 1:** Affiliation of Tobacco 21 case study participants from 10 states (N = 78), November 2018–March 2020.

Category	No.	(%)
National advocacy *	26	(33%)
Regional advocacy	27	(35%)
Health department staff	12	(15%)
Policymakers	11	(14%)
University affiliates/researchers	2	(3%)
Total	78	(100%)

* Note: National advocacy participants included individuals from the American Heart Association, American Lung Association, American Cancer Society, Campaign for Tobacco Free Kids, and Preventing Tobacco Addiction Foundation.

**Table 2:** Summary of state-level facilitators and barriers to state Tobacco 21 laws.

State	Passage Date/ Effective Date	Facilitators			Barriers		
		Policy Diffusion	Policy Entrepreneurs	E-cigarette Epidemic	Tobacco Industry Influence	Lack of Priority	Bill Language Controversy
California	05/04/16 06/09/16	N/A	Legislators	Legislators were starting to see signs of the e-cigarette epidemic.	Industry influenced legislators through campaign contributions and quid pro arrangements.	N/A	Health advocates unsuccessfully lobbied against military exemption. They were willing to withdraw support if preemption was in the bill.
New Jersey	07/21/17 11/01/17	Minimal local traction, primarily one high profile city.	Legislators	Minimal local traction, primarily one high profile city. N/A Influenced by NY, HI, CA passing T21.	Industry publicly testified against the bill.	N/A	N/A
Massachusetts	07/27/18 12/31/18	Extensive local traction; 80% of municipalities passed. Friendly competition with NYC passing.	Physicians and Legislators	Surge of JUUL use alarmed legislators and school administrators.	Industry posed public opposition through public hearings and advertisements.	Health advocacy groups focused on flavor bans. Legislators seen as removed from realities of youth smoking.	Advocates fought the inclusion of preemption in the final bill but were unsuccessful.
Virginia	02/21/19 07/01/19	N/A	Legislators	Legislators were motivated to prevent addiction and JUUL use.	Bill was strongly supported by Industry, and some evidence that they wrote it and proposed it to legislators at the last minute to minimize opportunity for opposition.	Clean Indoor Air policy was the priority for tobacco advocates at the time T21 was introduced.	Since the bill was introduced at the end of legislative session, advocates had little time to fight the weakest elements of the bill and were unsuccessful in having them removed from the final bill.
Vermont	05/16/19 09/01/19	Local traction in one influential city contributed momentum to other local action and the state bill. Encouraged by growing number of states passing T21, including nearby NY and MA.	Physician and Legislators	Increasing use of e-cigarettes by youth in schools could no longer be ignored by legislators.	Industry lobbied against the T21 bill.	N/A	Advocates fought against instituting penalties for possession and purchase and were ultimately unsuccessful.
Arkansas	03/28/19 09/01/19	No local traction. Legislators were influenced by a handful of other states that had passed.	Physicians and Legislators	Vaping epidemic alarmed the public and increased news media attention, which influenced legislators.	Industry lobbied extensively in support of weak legislation.	There were competing demands for the attention of health advocacy groups and legislators, notably the opioid epidemic.	Advocates fought the inclusion of preemption and military exemption but failed to keep them out of the final bill.
Texas	06/07/19 09/01/19	Local traction in one influential city was viewed as litmus test for state bill. Legislators motivated by the sense of T21 being a nationwide movement.	Legislators	The rise in vaping created more political will; legislators were influenced by increased calls from concerned constituents.	Industry lobbied against T21 and promoted messaging about e-cigarettes as harm reduction.	N/A	Some advocates withdrew support for T21 in the end because law included preemption, military exemption, and weak retailer penalty structure.
Connecticut	06/18/19 10/01/19	Local traction in two influential cities created momentum for other local action and the state bill.	Legislators	The increase in youth vaping along with calls from constituents motivated legislators.	Industry was first in opposition but changed position and negotiated with legislators.	N/A	N/A
Washington	04/05/19 01/01/20	Local traction in the capital contributed to statewide momentum. Also priding itself as a progressive state, so followed lead of HI passing.	Legislators	Local traction in the capital contributed to statewide momentum. Also priding itself as a progressive state, so followed lead of HI passing. Concerns about increased e-cigarette use among high schoolers increased legislative support.	Industry was initially in opposition and purportedly funded legislators to oppose the bills. Later in the campaign, industry switched positions and provided support for T21.	Tobacco control advocates were initially prioritizing other policies (e.g., taxation, smoke free air laws and fully funded tobacco program).	Advocates unsuccessfully fought the inclusion of preemption in the final bill, as well as a military exemption.
Utah	03/25/19 07/01/20	Industry supported T21 and played a role in promoting a weak bill.	Legislators	Local traction in two influential cities created momentum and rationale for the state bill.	Statements from the FDA and CDC declaring an epidemic attracted attention from legislators.	N/A	Advocates unsuccessfully fought several elements of the weak bill, including preemption, military exemption, grandfathering, and PUP penalties.

Note: N/A = Not applicable; preemption occurs when, by legislative or regulatory action, a higher level of government (state or federal) eliminates or reduces the authority of a lower level over a given issue [28].

**Table 3:** Facilitators and barriers to passing T21 laws.

Theme		Representative quotes
Facilitators	Policy Diffusion	“And I think there was a lot of concern among state legislators that we were going to have just a very non-uniform policy, that certain cities would be at the current age of 19 and other cities would start passing their own Tobacco 21 regulations, and I think there were some in our legislature who didn’t want those different policies. They didn’t want to have different policies across the state.” (UT, National advocacy) “But the interesting part is – what I discovered about Massachusetts – I don’t think they care so much about what the percentage of the population is under a [T21] regulation; they cared about the percent of towns, and things didn’t move as well statewide until we had 50 percent of the towns, which means we had to go into eastern Mass – western Mass, as well, and kind of get people. And the philosophy was get any town, no matter how big or how small.” (MA, Regional advocacy) “Well, and I also think there’s kind of always the healthy competition between states. I work both in Washington and Oregon, and any time you have one of those states that passes a policy that’s well received that’s supported by both sides of the aisle, I think the legislators on the other side of the state line will take a look at that. And Hawaii’s just across the ocean a couple thousand miles. So, I think it was also part of some growing momentum that was happening around the Pacific Northwest at the time as well.” (WA, National advocacy)
	Policy Entrepreneurs	“And what they [physician champions] were doing was with their white coats, it was incredibly powerful, and they were getting into the communities we hadn’t been in in a while, that we hadn’t been able to move a tobacco agenda forward in.” (MA, Health departments) “[W]e also had really, really great champions...Our attorney general is an unapologetic Democrat. And so, working with somebody like [Republican] Representative, who I would say, probably disagrees with us on 80 percent of everything else, but when it came to healthcare issues, he was just incredible. And I – that has a lot to do with it...I think that that was just very, very helpful.” (WA, Policymaker) “We had the Republicans carry the bill in both chambers and I think we have some Democratic lawmakers that have been tobacco control champions for a long time but they understand that having a Republican carry the goal is likely to be a more effective strategy...And so, having Republicans carry the bill...help[ed] work behind the scene as well as other Republicans also.” (TX, National Advocacy)
	Rise of E-cigarette Epidemic	“I think the teen vaping thing, the epidemic – it was alarming and it freaked people out…I bought a JUUL device and took it to the capitol and I was showing it to these people. And I was like, one in five kids in Arkansas are smoking this. It’s not healthy. It’s nicotine. They get hooked on it. And I had all the facts and stuff, and then you have the surgeon general’s warning – not only was that surgeon general warning a big deal, but it also created it where it put this JUUL-ing teen epidemic on their radar.” (AR, National advocacy) “We also had stories coming out from principals and administrators about bags and bags of e-cigarettes they had confiscated from students. They were using them in classes, in the hallways. They were using them in bathrooms. And the administrators and parents were really having a hard time figuring out how to keep these things out of schools because there is a lot of – it’s almost like students started using e-cigarettes and posting on Snapchat. Using them in the most risqué places. So, using them in classrooms and kind of getting those cool cred points with their friends for doing things like that. And so, it really has become part of the culture in a lot of our middle and high schools. And so, I think those kinds of stories definitely helped our legislators understand the urgency of addressing this problem.” (WA, National advocacy) “And that was, I think, also a very powerful message to have young people talk about what they were seeing in their high schools, having their bathrooms closed because people are using it so much, or saying – telling the stories about their friends who were getting these products from their brothers and sisters and using them and having to see them struggle with that. And so, I think youth involvement...was incredibly important, I think, for the decision makers and lawmakers to hear kids tell them what they’re seeing.” (CT, National advocacy)
Challenges	Tobacco Industry Influence	“I think Altria was trying to get in front... they’re pushing this weak bill across the nation and I think they were just trying to look like they were doing good for the youth as it related to tobacco products.” (VA, Health Departments) “I would definitely say it was the e-cigarette industry and the influence of the tobacco industry just in general. I mean they hired – I think we counted maybe 30, maybe 40 tobacco lobbyists in 2017. So if they have money, they have the influence to hire a lot of people to try to kill the bills that we’re trying to push forward.” (TX, National advocacy) “Well, I would say [one of the biggest challenges was] the industry, at every turn… and then, certainly, I would say JUUL. The tobacco industry, JUUL and the local vape industry. Because they are well funded. They have a lot of resources. And really, up until this last year, they were heavy, heavy opposition...[T]hey would never oppose in the hearings. They are busy working legislators. They can make hefty contributions.” (WA, Regional advocacy)
	Lack of Priority	“[A]s an evidence-based organization, we only take positions on policies after we have data and research...And when it came to Tobacco 21, we could assume that it would help, but we don’t operate on assumptions…[T]he Institute of Medicine report came out shortly after our legislative session ended. And that gave ACS CAN and some of other national advocacy partners the data and the research that we needed to really take a position of support on Tobacco 21….[T]he first year of the campaign, we were not supportive. Second year, it was one of our top legislative priorities.” (WA, National advocacy) “We would have much preferred to strengthen our clean air legislation and create more comprehensive clean indoor air policies across the state prior to Tobacco 21 being able to successful as we saw it.” (VA, Health departments) “[I]n the northeast where we’ve made a lot of progress [in tobacco control], people felt like we had done enough, and did we really need to do this? So the sense of urgency at times wasn’t – was a factor [and] a barrier for us.” (MA, Policymaker).
	Controversy over Bill Language	“We ended up having to put in a military exemption, not because anybody liked it or – the bill authors didn’t even support it – but the governor’s office was threatening to potentially veto the bill if it didn’t have a military exemption in it.” (TX, National advocacy) “I’ll say the final product was something that we didn’t support at all and we had problems with it prior to that, but – and the final product had a lot of challenges with it including there was a military exemption in the final product and new tobacco sales – the preemption of tobacco sales laws that was added to the bill, too. And I mean, from the [Organization’s] perspective we testified a few times in committee on some of the weaknesses in the bill. We – and we otherwise worked with – talked with legislators and also worked closely with our partners as well, too.” (AR, Regional advocacy) “[T]hat was yet another concern about the bill, was that it exempted the military. And I think, to a large extent, the – our advocacy groups, they wanted to kill the whole bill...And I’m like, you’re not gonna kill it, the governor supports it...it is clearly getting traction – bipartisan traction, you’re not going to be able to kill it, it’s not gonna happen, let’s at least move it from an F to a D by fighting the military exemption... They did not agree with my recommendation. They continued to fight the whole bill.” (VA, Health Department)

## Data Availability

The data presented in this study are available on request from the corresponding author. The data are not publicly available due to potential compromise of participant privacy.
